# Production of Raw Starch-Digesting Amylolytic Preparation in *Yarrowia lipolytica* and Its Application in Biotechnological Synthesis of Lactic Acid and Ethanol

**DOI:** 10.3390/microorganisms8050717

**Published:** 2020-05-12

**Authors:** Aleksandra Gęsicka, Monika Borkowska, Wojciech Białas, Paulina Kaczmarek, Ewelina Celińska

**Affiliations:** Department of Biotechnology and Food Microbiology, Poznan University of Life Sciences, ul. Wojska Polskiego 48, 60-627 Poznan, Poland; olka.gesicka@gmail.com (A.G.); monika.borkowska@up.poznan.pl (M.B.); wojciech.bialas@up.poznan.pl (W.B.); paulis.kaczmarek@gmail.com (P.K.)

**Keywords:** raw starch, *Yarrowia lipolytica*, heterologous protein production, protein expression platform, raw starch digesting enzymes, lactic acid, ethanol, *Kluyveromyces marxianus*, *Lactobacillus plantarum*

## Abstract

Sustainable economy drives increasing demand for raw biomass-decomposing enzymes. Microbial expression platforms exploited as cellular factories of such biocatalysts meet requirements of large-volume production. Previously, we developed *Yarrowia lipolytica* recombinant strains able to grow on raw starch of different plant origin. In the present study, we used the most efficient amylolytic strain as a microbial cell factory of raw-starch-digesting (RSD) amylolytic preparation composed of two enzymes. The RSD-preparation was produced in fed-batch bioreactor cultures. Concentrated and partly purified preparation was then tested in simultaneous saccharification and fermentation (SSF) processes with thermotolerant *Kluyveromyces marxianus* for ethanol production and *Lactobacillus plantarum* for production of lactic acid. These processes were conducted as a proof-of-concept that application of the novel RSD-preparation supports sufficient starch hydrolysis enabling microbial growth and production of targeted molecules, as the selected strains were confirmed to lack amylolytic activity. Doses of the preparation and thermal conditions were individually adjusted for the two processes. Additionally, ethanol production was tested under different aeration strategies; and lactic acid production process was tested in thermally pre-treated substrate, as well. Conducted studies demonstrated that the novel RSD-preparation provides satisfactory starch hydrolyzing activity for ethanol and lactic acid production from starch by non-amylolytic microorganisms.

## 1. Introduction

Starch is one of the most abundant carbohydrates, second only to cellulose, found in higher plant biomass. This homopolysaccharide is composed solely of glucose, and as such can be completely hydrolyzed to fermentable sugars without toxic by-products formation. Owing to its abundance, the desirable composition and high carbon content, starch is commonly used as a feedstock in biotechnological production of valued biomolecules, such as ethanol or lactic acid [1,2,3]. Valorization of starch-containing waste streams produced in massive amounts from confectionery manufacturing and bakeries, as well as discarded, damaged, or out of date products that return on site, were recently indicated as a largely ignored trend, with a huge potential for bioprocessing [2,4]. Up to date, these waste streams have been valorized through composting, as animal feed or disposed in landfills, and could be used as sustainable resource in microbial production of high-value added products.

Typical starch-based bioprocesses consist of two main steps: (i) starch liquefaction and saccharification, followed by (ii) conversion of glucose released from biopolymer to a target product. At first, the starch-containing feedstock is gelatinized by thermal treatment and then liquefied by thermostable α-amylase at 105 °C, and after cooling down to 60 °C, liquefied starch is saccharified to fermentable sugars by glucoamylase [5], which can be further converted to desired biomolecules. This initial thermal treatment generates high energy consumption, largely increasing the overall process cost and market price of the final product [6,7,8]. It has been estimated that energy demand of the pre-cooking step is equivalent to 30–40% of total process costs [9]. Hence, reducing the energy consumption is needed to make the starch-based processes more economically feasible. To this end, novel biocatalysts capable of digesting raw, non-pre-treated starch at lower temperatures are being sought [10].

The application of raw starch degrading enzymes (RSDE) able to hydrolyze granular non-pre-treated starch below the liquefaction temperature can bring significant reduction in the energy consumption compared with the conventional processes [10,11]. As evidenced by Robertson et al. [11], exploitation of RSDE in ethanol production from starch reduces the energy input equivalent to 10–20% of the fuel value. RSDE exploitation is a pre-requisite for conducting starch-based bioprocesses according to simultaneous saccharification and fermentation (SSF) strategy, relying on provision of both RSDE and thermotolerant microorganisms to the same fermentation vessel at the same time. Upon the SSF both catalysts act concurrently, which brings many advantages, like improved efficiency of saccharification by avoiding end-product inhibition, decreased investment cost and number of unit operations, and reduced cooling costs, as the whole process is carried out at a temperature between 30 and 50 °C [12,13,14].

Typical RSDE preparations are composed of α-amylase and glucoamylase working synergistically to rapidly and completely hydrolyze raw starch into the fermentable sugars. The amylases having RSD activity can be found in many organisms, including plants, animals and microorganisms, however only the latter can be reasonably exploited in RSDE production [10,15]. Currently, the RSDE available on the market are mainly produced by fungal species, such as *Aspergillus* sp., *Penicillium* sp. and *Rhizopus* sp. [16]. As the demand for RSDE is increasing, new RSDE with high enzymatic activity, desired substrate specificity and characteristics are being sought. 

Apart from the native, fungal producers mentioned above, efforts are being pursued towards development of microbial cell factories being able to produce RSDE of desired characteristics [17,18,19]. Heterologous production of proteins offers several key advantages, like controlled synthesis without the necessity of starch as an inducer, high overexpression enhanced by engineered regulatory elements, less accompanying proteins from engineered microbial host, easier purification of the target enzymes or ease of manipulation in scaled-up processes, just to name a few. Amongst a variety of microbial expression platforms, those with efficient secretory pathway are particularly suited for production of extracellular proteins, frequently requiring complete maturation and post-translational modifications. While *Komagataella phaffi* (*Pichia pastoris*) is the most popular yeast workhorse in this regard, increasing evidence shows that *Yarrowia lipolytica* is a highly attractive alternative. Suitability of *Y. lipolytica* to be an industrial workhorse is repeatedly proved [20,21,22]. Recently, it has been elegantly demonstrated that upon direct comparison, *Y. lipolytica* outperforms *P. pastoris* in terms of heterologous secretory protein production [23]. Its ability to grow to high-cell density [24], resistance to environmental stresses encountered in the industrial context [25], and highly efficient secretory pathway [26] account for *Y. lipolytica* high performance in the heterologous secretory protein production.

Therefore, in this study we exploited *Y. lipolytica* expression platform for heterologous production of amylolytic preparation composed of two enzymes produced simultaneously. To this end, we cloned and expressed RSD α-amylase from rice weevil (*Sitophilus oryzae*) (SoAMY) and glucoamylase from fungi *Thermomyces lanuginosus* (TlGAMY) in *Y. lipolytica* fed-batch bioreactor cultivations. A simple procedure was used to formulate ready-to-use amylolytic preparation, and its yield was analyzed. Finally, the obtained preparation was tested in two proof-of-concept SSF processes: (i) production of ethanol by thermotolerant yeast *Kluyveromyces marxianus* and (ii) synthesis of lactic acid by *Lactobacillus plantarum.* Both these strains lack native ability to hydrolyze starch, which was used as the main carbon source; so, the processes completely relied on action of the amylolytic preparation. The target molecules and proof-of-concept processes were selected based on high market demand for the two compounds. Bioethanol is commonly used as an alternative fuel and is thus in high continuous demand. Lactic acid is mainly applied in cosmetics industry and as a platform for the production of green solvents, fuel precursors and biodegradable plastic PLA (polylactic acid) [3,27].

## 2. Materials and Methods 

### 2.1. Strains and Basic Culturing Media

The genes encoding heterologous alpha-amylase and glucoamylase were cloned in *Y. lipolytica* Po1h strain (*MatA, ura3-302, xpr2-322, axp1-2*), according to previously described methodology [28]. Both genes were cloned under a strong, growth phase-dependent promoter p4UASpTEF, in a double transcription units-bearing expression cassette, assembled via Golden Gate strategy [29] with *TlGAMY* gene in the first transcription unit and *SoAMY* gene in the second transcription unit. Then, both genes were transcriptionally fused to a signal peptide of YALI0E22374g [30] at 5’ end (previous denomination SP3), and to 6His-Tag at 3’ end, to facilitate the protein purification. The resultant recombinant strain GGY215 was used as the production platform in this study. The strain was stored as glycerol stock in 15% glycerol at −80°C. Prior to each culture run, the strain was streaked on YPD agar plate ((g/L): YE (BIOCORP), 10, Peptone (BIOCORP), 20, glucose (POCh), 20, agar (BTL), 15) and incubated at 30 °C for 48 h.

*Kluyveromyces marxianus* DSMZ 5422 was purchased from DSMZ (German Collection of Microorganisms and Cell Cultures). The strain was used in a proof-of-concept process of ethanol production from raw starch. The strain was confirmed to be unable to decompose starch through iodine drop test (agar plate, (g/L): rich broth, 15 (Biomaxima, Lublin, Poland), agar, 20, starch, 10), as described previously [31]. The strain was maintained as glycerol stock in 15% glycerol at −80 °C. Prior to each culture run, the strain was streaked on a YPD agar plate and incubated at 30 °C for 48 h.

*Lactobacillus plantarum* strain is an own isolate, deposited in the Collection of Department of Biotechnology and Food Microbiology, Poznan University of Life Sciences, Poland. The strain was used in a proof-of-concept process of lactic acid production from starch. The strain was confirmed to be unable to decompose starch through iodine drop test, as described above for *K. marxianus*. The strain was maintained as glycerol stock in 15% glycerol at −80 °C. Prior to each culture run, the strain was streaked on a MRS agar plate ((g/L): ready mix, 62; Oxoid) and incubated at 30 °C for 48 h.

### 2.2. Fed-Batch Bioreactor Cultivations

The production strain *Y. lipolytica* GGY215 was transferred from fresh YPD agar plate into 200 mL of YPG20 medium ((g/L): YE, 10, Pepton, 20, glycerol (POCh), 20) and cultured over 22 h at 30 °C with shaking 250 rpm (BIOSAN, ES-20, Riga, Latvia). Such a pre-culture was used for inoculation of 1.8 L YPG100 production medium ((g/L): YE, 10, Pepton, 20, glycerol, 100) in Biostat B plus bioreactor (Sartorius Stedim, Goettingen, Germany), with a total volume of 5 L and a working volume of 2 L (after inoculation)–2.5 L (after feeding). The bioreactor was equipped with two Rushton turbines attached to a stirrer shaft, and the pH, temperature and dissolved oxygen probes. Temperature, pH, vvm and pO2 were maintained through the culturing time at 28 °C, 5.5, 2, 20%, respectively. pO2 was stabilized by activating cascade pO2-stirring and manual control of air flow. When the initial portion of glycerol was consumed (at ~ 24 h), 0.5 L of 5-fold concentrated YPG100 medium was fed into the culture. The cultures were continued for 72 h. Samples were collected in time intervals, centrifuged for 5 min at 12,045× *g* (Eppendorf, MiniSpin, Hamburg, Germany), and biomass and supernatant were stored separately at −20 °C, until analyzed. Samples were analyzed for concentration of dry biomass (gravimetric method), substrate and metabolites (HPLC) and amylolytic activity in the supernatant (microSIT). The production cultures were run in 4 independent batches.

### 2.3. Formulation of SoAMY-TlGAMY Preparation

The total post-culturing suspension (approx. 2.5 L) was centrifuged for 1 h at 5063× *g* in +4 °C (Hettich, Rotanta, Tuttlingen, Germany). The biomass was discarded, and the supernatant was subjected to protein precipitation with ammonium sulfate. Ammonium sulfate (POCh) was added in portions to reach the final saturation of 80%. The solution was slowly mixed overnight at +10 °C. Then, the solution was centrifuged for 2 h at 5063× *g* in +4 °C (Hettich, Rotanta, Tuttlingen, Germany). Samples of the supernatant were stored at −20 °C for analysis. The deposit was resuspended in sterile 0.1 M acetate buffer pH 5.5 and filtered through 0.45 µm low protein retention filter (Millex, Millipore, Burlington, MA, USA). Subsequent batches of such SoAMY-TlGAMY preparation were standardized based on determination of amylolytic activity through microSIT assay. Such crude preparation was used in the proof-of-concept processes.

### 2.4. Purification of SoAMY-TlGAMY and Analysis

To analyze the process of SoAMY-TlGAMY preparation formulation in terms of the enzymes yield and losses, the heterologous enzymes were purified from a control culture to apparent homogeneity, using affinity chromatography, according to a procedure described previously [32]. Briefly, the ammonium sulfate-precipitated proteins were resuspended in a binding buffer (phosphate buffer, 20 mM, pH 7.4; NaCl, 0.5 M; imidazole, 20 mM), filtered through 0.45 µm syringe filter (Millex, Millipore, Burlington, MA, USA), and loaded onto the ÄKTA FPLC system (ÄKTA Pharmacia GE FPLC; Chicago, IL, USA) equipped with an IMAC HisTrap HP column (5 mL, GE Healthcare). Increasing gradient of the elution buffer (phosphate buffer, 20 mM, pH 7.4; NaCl, 0.5 M; imidazole, 0.5 M) was applied at the flow rate of 5 mL/min. Fractions flow-through (F-T), wash-unbound (W-U) and elution fractions (F1, F2), showing increased signal at 280 nm wavelength, were subjected to concentration and buffer exchange using Amicon columns with 3 kDa cut-off point (Millipore, Burlington, MA, USA). These fractions, together with the supernatant and resuspended deposit after the ammonium sulfate precipitation, and the culture supernatant, were subsequently subjected to: (i) SDS-PAGE according to a standard Laemmli method [33]; the proteins were resolved in 15% denaturing polyacrylamide gel (BioRad system, Hercules, CA, USA); (ii) microBCA for determination of protein concentration (SigmaAldrich kit, St. Louis, MO, USA); (iii) microSIT assay for determination of amylolytic activity, according to [34].

### 2.5. Proof-of-Concept Process 1: EtOH Production by K. marxianus in SSF Process with SoAMY-TlGAMY Crude Preparation

#### 2.5.1. Shake-Flask Production Cultures

*K. marxianus* DSMZ 5422 was transferred from a fresh YPD agar plate into 25 mL of premedium ((g/L): YE, 3, malt extract (ME; bioMérieux Poland, Warsaw, Poland), 3, Peptone, 5, glucose, 10) and cultured over 22 h at 30 °C with shaking 250 rpm. In total, 40 mL of production medium in 100 mL SIMAX bottles ((g/L): native raw starch, 40, YE, 3, ME, 3, KH_2_PO_4_ (POCH), 1, CaCl_2_ (POCH), 0.26, chloramphenicol, 35 µg; pH 5.5) were inoculated at 10% with the preculture. Native rice starch was added to sterilized medium, directly before inoculation, to avoid starch gelatinization. The crude preparation was added into the medium in two dose variants: at 20 and 25 AU per gram of starch, at the moment of inoculation. The SSF process was conducted in a rotary shaker-incubator with shaking at 250 rpm and temperature of 32, 36 and 40 °C for 54 h. Control runs with native starch and the enzymatic preparation, but without the strain, were conducted under corresponding conditions (temperature and doses) in biological triplicate. Samples were collected in time intervals and stored at −20 °C. Ethanol and residual glucose concentrations was monitored throughout the experiment through the HPLC technique. The cultures were carried out in three biological replicates.

#### 2.5.2. Bioreactor Production Cultures

The SSF cultures were conducted in Minifors 2 bioreactors (Infors HT, Bottmingen-Basel, Switzerland) with a total volume of 2.6 L and a working volume of 0.5 L. The bioreactor was equipped with two Rushton turbines attached to a stirrer shaft, pH, temperature and dissolved oxygen probes. Temperature, pH and stirring were maintained through the culturing time at 36 °C, 5.5 and 200 rpm respectively. Three different aeration strategies were tested; IS—air provided through a sparger immersed in the culture, air flow of 0.04 vvm throughout the culture, HS–air provided from a headspace, air flow of 0.04 vvm throughout the culture, IS21—air provided through a sparger immersed in the culture, air flow of 0.5 vvm only for the first 21 h of culturing. Raw starch was added to the medium after bioreactor sterilization to avoid gelatinization. The crude amylolytic preparation was added at the amount of 20 AU per gram of starch at the moment of inoculation (at 5% with preculture developed as indicated in 2.5.1).

To assess efficiency of SoAMY-TlGAMY crude preparations, three control cultures were conducted: (i) with glucose instead of starch as a substrate, (ii) with application of commercial RSDE preparation STARGEN 002 at the same dose of 20 AU per gram of starch, (iii) SHF (separate hydrolysis and fermentation) process: starch hydrolysis for 6 h at 40 °C preceding the inoculation. The control processes were conducted under HS aeration strategy. All the processes were carried out for 72 h. Samples were collected in time intervals and stored at −20 °C. Ethanol and residual glucose concentration was monitored throughout the experiment through the HPLC technique. Microbial growth was assessed through determination of viable counts.

### 2.6. Proof-of-Concept Process 2: LA Production by Lb. Plantarum in SSF Process with SoAMY-TlGAMY Crude Preparation

#### 2.6.1. Shake-Flask Production Cultures

*Lb. plantarum* precultures were developed from glycerol stocks by twice subculturing the strain and increasing volume of MRS liquid medium (MRS mixture (Oxoid, Hampshire, United Kingdom), 52 (g/L)) for 18–20 h at 30 °C without shaking. The flask production cultures were conducted in 40 mL of the production medium in 100 mL SIMAX bottles ((g/L): native raw starch, 20, YE, 10, ammonium citrate (Sigma-Aldrich, St. Louis, MO, USA), 2, K_2_HPO_4_ (POCH), 2, sodium acetate (Chempur, Piekary Slaskie, Poland), 5, MgSO_4_ × 7 H_2_O, 0.2, MnSO_4_ × 4 H_2_O (Enola, Riga, Latvia), 0.05). The starch was added either prior to sterilization (cooked starch variants) or prior to inoculation (raw starch variants). The media with cooked starch were mixed directly after sterilization on a magnetic stirrer to avoid solidification of medium for at least 1 h. The production cultures were inoculated with 2% of the preculture. The SoAMY-TlGAMY crude preparation (60 AU per gram of starch) was added into the medium at the moment of inoculation. The SSF processes were carried out for 72 h at 30 °C without shaking. Control runs with raw starch and the enzymatic preparation, but without the strain were conducted under corresponding conditions (temperature and dose) in biological triplicate. Samples were collected in time intervals and stored at −20 °C. Lactic acid and residual glucose concentration was monitored throughout the experiment through the HPLC technique. The cultures were carried out in three biological replicates.

#### 2.6.2. Bioreactor Production Cultures

The SSF cultures were conducted in Minifors 2 bioreactors with a total volume of 2.6 L and a working volume of 0.5 L. The bioreactor was equipped with two Rushton turbines attached to a stirrer shaft, pH and temperature probes. Temperature, pH and stirring were maintained through the culturing time at 30 °C, 6.5 and 50 rpm respectively. pH level was established, based on previous literature reports [35,36]. The production medium composition was described in point Section 2.6.1. In the case of the cooked starch variants, after sterilization, the medium was stirred in a bioreactor at 500 rpm overnight. The SoAMY-TlGAMY crude preparation was added at the dose of 60 AU per gram of starch. The bioreactor was inoculated at 5% with preculture developed as indicated in Section 2.6.1. The processes were carried out for 72 h. Samples were collected in time intervals and stored at −20 °C. Lactic acid and residual glucose concentration was monitored throughout the experiment through the HPLC technique. The microbial growth was assessed through determination of viable counts.

### 2.7. Analytical Methods

#### 2.7.1. Amylolytic Activity Assay (microSIT)

Amylolytic activity of the SoAMY-TlGAMY preparations was analyzed using microSIT procedure, described earlier [34]. Rice starch solution (2 mg/mL) in acetate buffer (100 mM, pH 5.0) was used as a substrate. The reaction was initiated by adding equal volume of the enzyme-containing samples, and continued by incubation at 40 °C. The reactions were stopped by adding 0.5 volume of 1 M HCl and stained with iodine solution (5 mM I2 in 5 mM KI) added at equal amount. The amylolytic activity was determined based on absorbance at 580 nm in MTP(microtiter plate)-reader (TECAN, Infinite M200 automatic plate reader; Tecan Group Ltd., Männedorf, Switzerland). One AU was defined as the amount of an enzyme that contributes to decrease in starch-iodine staining value equivalent to 1 mg of starch during 1 min.

#### 2.7.2. Protein Concentration Assay (microBCA)

Concentration of total proteins contained in the samples was measured using BCA protein assay kit (Sigma Aldrich, St. Louis, MO, USA), in a miniaturized form. Standard curve was prepared with a standard BSA solution, ranging from 400 to 1000 µg per mL. All the analyzed samples were first subjected to buffer exchange (20 mM phosphate buffer), to remove interference with incompatible compounds, and diluted to fit within the standard curve range. The reactions were conducted according to the manufacturer’s specifications in technical triplicate.

#### 2.7.3. Determination of Compounds Concentration—HPLC

##### Glycerol and Metabolites Concentration

Concentration of glycerol, erythritol, mannitol, citric acid, alpha-ketoglutarate, ethanol and lactic acid was determined using the following HPLC methodology. Samples withdrawn from the cultures were centrifuged (3 min, 24,652× *g*), 5x diluted in distilled water and passed through a 0.45 µm filter (Millex, Millipore, Burlington, MA, USA). An Agilent Technologies 1200 series chromatograph, equipped with Rezex ROA 300 × 7.80 mm (Phenomenex), autosampler (G1329B), double pump (G1312B), refractic index detector (G1362A) and diode array detector (G1315C), was used for the analyses. Also, 0.005N H_2_SO_4_ was used as the eluent at 0.6 mL/min, under isocratic conditions. The analysis was conducted at 40 °C. In total, 10 µL of the samples were loaded onto the column. Quantitative and qualitative identification of the compounds was carried out using external standards and the peak height (automatic determination and integration using ChemStation for LC 3D systems, Agilent, Santa Clara, CA, USA).

##### Starch-Decomposition Products Concentration (dp1-dp7)

Concentration of saccharides of polymerization degree from dp1 to dp7 was determined using the following HPLC methodology. Samples withdrawn from the cultures were centrifuged (3 min, 24,652× *g*) and passed through a 0.45 µm filter (Millex, Millipore, Burlington, MA, USA). Agilent Technologies 1200 series chromatograph, equipped with Rezex RSO-Oligosaccharide Ag + 200 × 10 mm (Phenomenex) column, autosampler (G1329B), double pump (G1312B), and refractic index detector (G1362A) was used for the analyses. H_2_O was used as the eluent at 0.3 mL/min, under isocratic conditions. The analysis was conducted at 80 °C. Then, 10 µL of the samples was loaded onto the column. Quantitative and qualitative identification of the compounds was carried out using external standards and the peak height (automatic determination and integration using ChemStation for LC 3D systems, Agilent, Santa Clara, CA, USA).

#### 2.7.4. Microbial Growth Analysis

##### 2.7.4.1. Gravimetric Method—Dry Cellular Biomass Determination

Where indicated, the biomass concentration in the culture samples was determined according to a standard gravimetric method. The biomass samples were first defrosted, washed twice in distilled water and dried at 105 °C until stable weight readout (typically 24–48 h).

##### 2.7.4.2. Spectrophotometric Measurement at 600 nm Wavelength (OD600)

Where indicated, the biomass concentration in the culture samples was determined using spectrophotometric measurements of absorbance at 600 nm wavelength. The biomass samples were prepared as described in point Section 2.7.4.1. Measurements were conducted using Analytik Jena Spectrophotometer (Analytik Jena, Jena, Germany), WinASPEKT Software and compatible, standard 1.5 mL PS cuvettes (Starstedt, Nümbrecht, Germany).

##### 2.7.4.3. Viable Counts

Where indicated, microbial growth was analyzed through determination of viable counts per culture volume unit. This technique was used for starch-containing samples, where neither dry cellular weight assessment nor spectrophotometric measurement were useful due to the substrate properties. Upon sampling, culture samples were immediately subjected to decimal dilutions in sterile saline solution. Samples were then surface plated on an agar-solidified medium (YPD or MRS, for *K. marxianus* or *Lb. plantarum*, respectively), and the plates were incubated at 30 °C or 37 °C for 24 or 48 h. Viable counts were expressed as cfu/mL of the culture sample.

### 2.8. Statistical Analysis and Data Managment

Obtained results were expressed as mean ± standard deviation (±SD) of the replicates described above and indicated in figure captions. Statistical importance of the differences between compared sets of data was analyzed using one-way analysis of variance (ANOVA) and Tukey’s multiple comparison tests (Statistica; Statsoft Poland Inc., Cracow, Poland). The levels of significance were set at *p* < 0.05 or *p* < 0.001 (indicated). Graphical presentation of the obtained data was done using Microsoft Excel 2013 software. Due to difficulties in determination of residual raw starch concentration in the samples from *K. marxianus* and *Lb. plantarum* cultivations, yield of ethanol and lactic acid were expressed as gram of the target molecule per gram of the substrate provided at the beginning of the culture, according to methodology and equation 3.69.a.b, page 102 provided in [37].

## 3. Results

### 3.1. Production and Purification of SoAMY-TlGAMY Enzymatic Preparation

**Figure 1:**
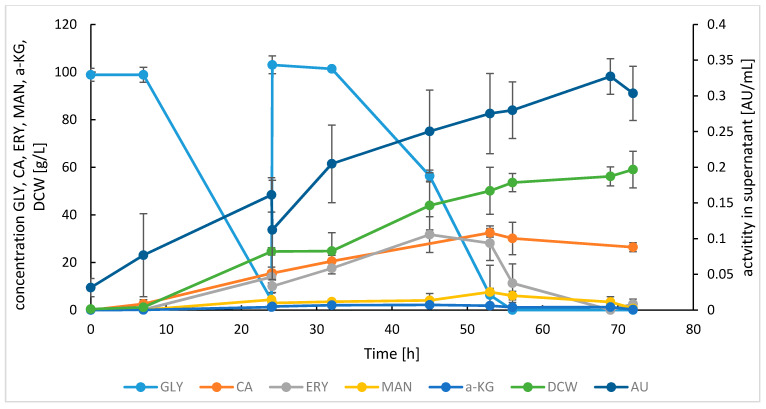
Time-course of SoAMY-TlGAMY proteins production, utilization of glycerol and synthesis of metabolites in fed-batch production cultures of *Y. lipolytica* GGY215. Letter codes: GLY, glycerol; CA, citric acid; ERY, erythritol; MAN, mannitol; A-KG, alpha-ketoglutarate; DCW, dry cellular biomass; AU, amylolytic activity in supernatant assayed by microSIT assay. Values indicate means ± SD from four independent runs.

Production of the SoAMY-TlGAMY enzymatic preparation was conducted in fed-batch bioreactor cultures of the *Y. lipolytica* GGY215 recombinant strain. The strain expresses the two heterologous genes under control of a strong promoter p4UASpTEF. Under the adopted experimental conditions, accumulation of SoAMY-TlGAMY-derived amylolytic activity in the culture supernatant reached 0.327 ± 0.025 AU/mL (Figure 1). Production of the target polypeptides was accompanied by high biomass growth (59.03 ± 7.67 g/L) and synthesis of small molecular weight metabolites typical for *Y. lipolytica* cultures under adopted conditions. Erythritol (ERY) and citric acid (CA) were the two dominant metabolites, reaching peak concentration of 31.68 ± 7.51 and 32.46 ± 1.78 g/L, respectively. The following decrease in ERY concentration is typical under carbon source limitation for *Y. lipolytica* cultures. 

**Figure 2:**
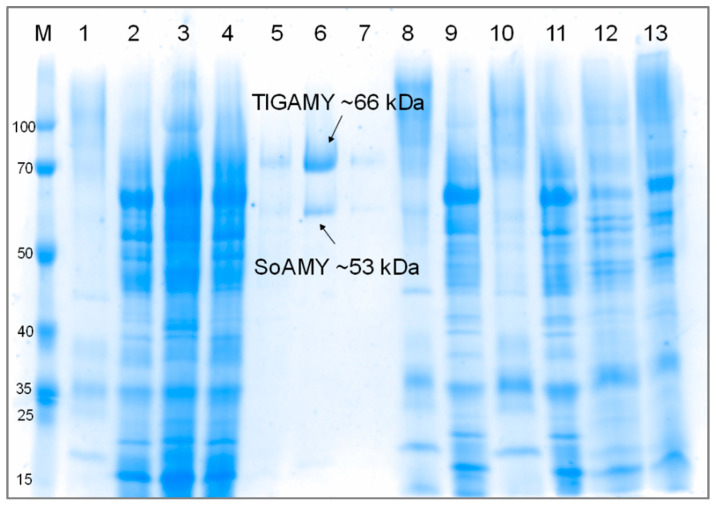
Electrophoretic separation of the protein fractions. Proteins were separated in 15% SDS-PAGE. M: PageRuler™ Prestained Protein Ladder (ThermoScientific, Waltham, MA, USA), 1, 8, 10: supernatant after ammonium sulfate precipitation, 2, 9, 11: resuspended protein after ammonium sulfate precipitation (crude preparation), 3: F-T, 4: W-U, 5: Fraction 1 (F1) with increased Abs280 during affinity chromatography, 6: F2 with increased Abs280 during affinity chromatography, 7: F3 with increased Abs280 during affinity chromatography, 12, 13: culture medium supernatant.

To formulate the crude SoAMY-TlGAMY preparation, the proteins contained in the post-culturing medium were subjected to the ammonium sulfate precipitation. To assess efficiency of such approach and to evaluate the degree of potential losses in SoAMY-TlGAMY, the consecutive protein fractions were analyzed for total protein concentration (microBCA), the protein profile through SDS-PAGE, and for total amylolytic activity (Figure 2, Table 1). Additionally, a control run followed by purification of SoAMY and TlGAMY proteins through the affinity chromatography was conducted. As shown in Figure 2, the two heterologous proteins were successfully expressed and secreted to the culture medium. The adopted ammonium sulfate precipitation procedure allowed to concentrate the proteins in the pellet, as they could be later recovered from the precipitate during the affinity chromatography.

According to data presented in Table 1, the adopted precipitation procedure was efficient in concentrating extracellular proteins in the ammonium sulfate-deposited pellet (10-fold). Importantly, this AS pellet fraction was enriched in SoAMY-TlGAMY proteins, as shown by the total amylolytic activity values (317.83 ± 21.57 vs. 0.18 ± 0.16 [AU/mL]) and the specific activity (8.01 ± 1.4 vs. 0.06 ± 0.05 (AU/mg)). Further purification by the affinity chromatography was on one hand coupled with high losses of SoAMY-TlGAMY polypeptides in F-T and W-U fractions, as evidenced by the amylolytic activity detected in these fractions (64.05 ± 0.92 and 328.94 ± 14.62 (AU/mL)) and the specific activity values (2.4 ± 0.03 and 80.65 ± 3.58 (AU/mg)); but on the other, led to highly concentrated F2 fraction of high total and specific activity (5609.29 ± 199.7 (AU/mL) and 13159.51 ± 468.49 (AU/mg)). As shown in Figure 2, this fraction contained SoAMY and TlGAMY as the only detectable polypeptides. The high losses, especially in W-U fraction, preclude exploitation of affinity chromatography in the current format as the down-stream purification operation. Since the ammonium sulfate precipitation was efficient in terms of SoAMY-TlGAMY concentration, and the bulk proteins did not inhibit amylolytic activity, further studies were conducted with the crude preparation-resuspended protein pellet after ammonium sulfate precipitation. Subsequent batches of the crude preparation were normalized with respect to their total activity (AU/mL), to provide desired amounts of AU per gram of starch provided to the process.

### 3.2. Proof-of-Concept Process 1: Ethanol Production by K. marxianus in SSF Process with SoAMY-TlGAMY

#### 3.2.1. Flask SSF Cultures

**Figure 3:**
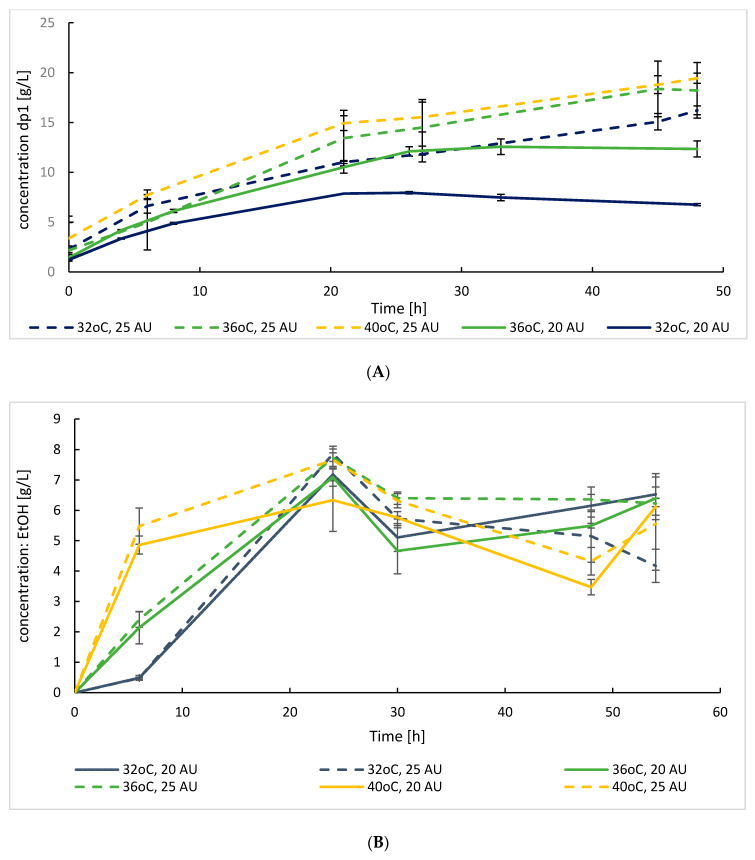

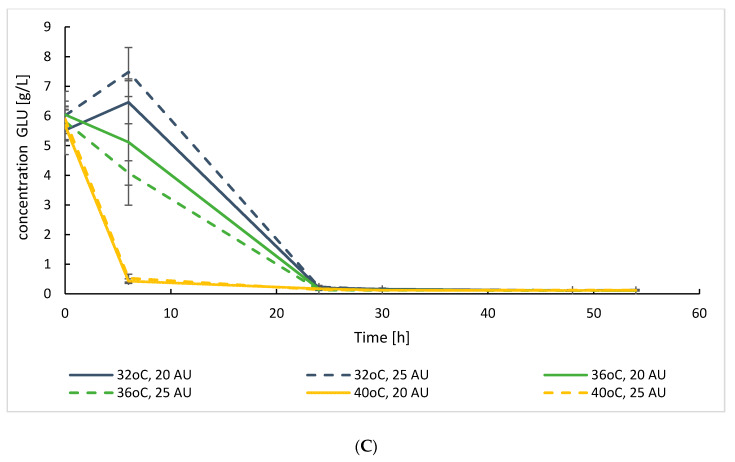
Time course of (**A**) dp1 release from raw rice starch by SoAMY-TlGAMY crude preparation at different doses and temperatures (control run without the strain). Time course of ethanol production (**B**) and residual glucose concentration (**C**) in *K. marxianus* DSMZ 5422 SSF process with two doses: 20 and 25 AU per gram of starch and at three temperatures: 32, 36 and 40 °C. Letter codes: EtOH, ethanol; GLU, glucose. Values indicate means ± SD from three independent runs.

The SoAMY-TlGAMY preparation was tested in a proof-of-concept process of bioethanol production from raw starch by *K. marxianus* strain. Initial SSF cultures were conducted in flasks at 32, 36 and 40 °C, with the preparation doses of 20 and 25 AU per gram of starch, to select optimal conditions. Additionally, the profile and time-course of (oligo)saccharides released from raw starch under these conditions was analyzed, within the range dp1-dp7 (Figure 3A). Action of the SoAMY-TlGAMY preparation on native rice starch led to decomposition of the biopolymer and only monosugar dp1 (glucose) was detected in the following HPLC analysis (within the range dp1-dp7). Saccharides from dp2 to dp7 were not detected in the analyzed samples. The results of ethanol production are shown in Figure 3B, and residual concentration of glucose is shown in Figure 3C.

As demonstrated in Figure 3A, both increase in the preparation dose by 5 AU (20 to 25 AU per gram of starch) as well as in the temperature of hydrolysis by 4 °C (from 32 to 36 °C) brought statistically significant improvement in dp1 release form starch (*p* < 0.001). The difference in monosugar release between 36 °C and 40 °C was not statistically significant (at *p* < 0.05). Based on the data presented in Figure 3B, it can be observed that at 40 °C the SSF process was more rapid than those conducted at 32 and 36 °C, as seen from nearly complete consumption of released dp1 and the highest ethanol titer (~5.17 ± 0.44 vs. 2.28 ± 0.19 g/L for 36 °C) at the early time-point (6 h). Nevertheless, the peak ethanol concentration was observed at 24 h of the cultures conducted at 36 °C reaching 7.09 ± 0.29 and 7.73 ± 0.29 g/L with ethanol yield of 0.177 vs. 0.193 g/g, respectively for 20 and 25 AU dose, which was concomitant with complete of glucose, initially released from starch (6 g/L; Figure 3C, Table 2). Further release of dp1 from raw starch was lower than its utilization rate by *K. marxianus*, as no residual glucose was detected. Decrease in ethanol concentration in terminal time-points depicted in Figure 3B results from its evaporation from the culture. Additionally, Figure 3C in 6 h time-point shows that at 32 °C dp1 release from starch is not well balanced with its consumption by microbial cells (more is released than consumed), which further indicates that this temperature is not suitable for the SSF process with *K. marxianus* as the ethanol producer, as it limits the microbe growth.

#### 3.2.2. Bioreactor SSF Cultures

**Figure 4:**
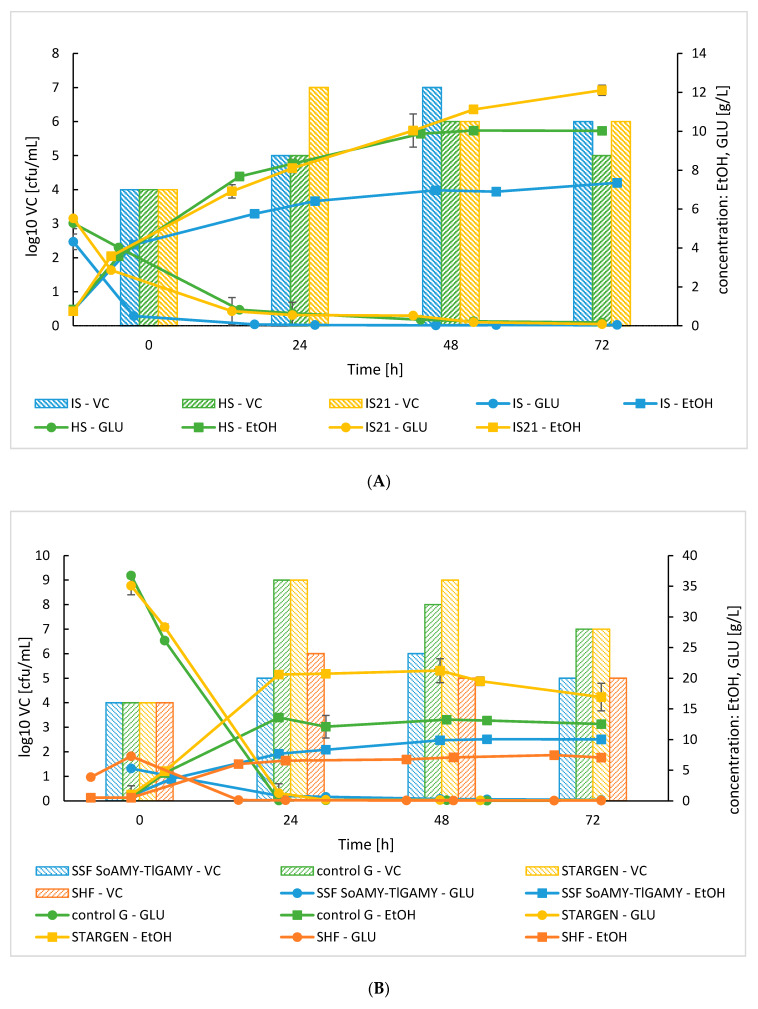
Time course of ethanol production and residual glucose concentration in *K. marxianus* DSMZ 5422 SSF processes. (**A**) Adjustment of aeration strategy: IS—air provided through a sparger immersed in the culture, HS—air provided from a headspace, IS21—air provided through a sparger immersed in the culture only for the first 21 h of culturing. (**B**) Comparison of SSF SoAMY-TlGAMY with control processes: using glucose as the carbon source (control G), using commercial STARGEN RSDE (raw starch digesting enzyme) at 20 AU per gram of starch, adopting SHF (separate hydrolysis and fermentation; two-step process with separate stage of 6 h hydrolysis of starch). Letter codes: EtOH, ethanol; GLU, glucose; VC—viable count. Values indicate means ± SD from two independent runs.

The initial bioreactor processes of ethanol production from raw starch in the SSF with the SoAMY-TlGAMY and the *K. marxianus* were focused on adjustment of the aeration strategy. To this end, the air was provided either through (i) a sparger immersed in the culture medium (IS), (ii) through its provision into head-space of the culture (HS), or (iii) through an immersed sparger, but only for the first 21 h of culturing (IS21; to promote biomass growth). Conducted statistical analysis confirmed that aeration strategy had a significant impact on ethanol production (*p* < 0.001). The ethanol yield (calculated for 24 h of culturing) ranged from 0.144 to 0.192 g/g (for IS and HS respectively), depending on aeration strategy (Table 2). As shown in Figure 4A, in all tested variants, the maximum production of ethanol was observed at 70 h of the process (vs. peak at 24 h in shake flask). During the first 48 h of culturing, the ethanol production in IS21 and HS processes was highly corresponding, and no statistically significant difference was observed between these processes (*p* < 0.001). Only at the terminal time-points, the IS21 process turned out to be more efficient (10.02 ± 0.19 vs. 12.11 ± 0.26 g/L; *p* < 0.001). On the other hand, IS process was less efficient (*p* < 0.001) in terms of ethanol production than HS and IS21 in earlier (48 h) and terminal (72 h) time-points, reaching maximally 7.35 ± 0.07 g/L. The most rapid growth of the cells was observed for IS21 cultures, equal to 10^7^ cfu/mL at 24 h, which was reached in IS processes at 48 h. The viable counts decreased in the following time-points, together with increasing ethanol concentration and limited oxygen provision in all the culturing variants.

To assess competitiveness of a process with the SoAMY-TlGAMY preparation, comparative cultures were conducted using the same major operation conditions (36°C, HS aeration, *K. marxianus*, raw rice starch, SoAMY-TlGAMY; individually exchanged where indicated), but differing in: (i) the substrate—glucose instead of starch (“control G”), (ii) the type of preparation—commercial RSDE instead of SoAMY-TlGAMY (“STARGEN”), (iii) the mode of operation—SHF instead of SSF (SHF) (Figure 4B). Importantly, all the implemented modifications had a statistically significant impact on the obtained ethanol concentration (*p* < 0.001). When glucose was used as the sole carbon source, the peak ethanol concentration reached 13.59 ± 0.27 vs. 7.68 ± 0.05 for control G and SSF SoAMY-TlGAMY variants, respectively, at 22 h, but the final ethanol concentration at 72 h was not statistically different between these two variants (at *p* < 0.05). The process with the SoAMY-TlGAMY preparation was importantly less efficient in terms of ethanol production than the process conducted with commercial enzymatic preparation STARGEN (*p* < 0.001). The use of commercial RSDE brought the highest ethanol concentration of all tested variants (21.22 ± 1.95 g/L). Concomitantly, the initial glucose concentration released from starch by STARGEN preparation was 7-fold higher than by SoAMY-TlGAMY (35.07 ± 1.47 vs. 5.28 ± 0.28 g/L); and in this regard, was similar to control G variant; which was also reflected by ethanol yields reaching 0.515, 0.340 and 0.173 g/g (at 24 h; for STARGEN, control G and SoAMY-TlGAMY, respectively; Table 2). High glucose utilization during the first 24 h of culturing in control G and STARGEN processes brought the highest cell growth, reaching slightly above 10^8^ cfu/mL. SHF process, preceded by 6 h hydrolysis of starch by SoAMY-TlGAMY preparation prior to inoculation, showed slightly higher initial glucose concentration compared to SSF SoAMY-TlGAMY process (7.27 ± 0.35 vs. 5.28 ± 0.28 g/L); but the final ethanol titer was not significantly higher in this culturing variant (7.06 ± 0.10 vs. 10.02 ± 0.19 g/L; not significant at *p* < 0.05).

### 3.3. Proof-of-Concept Process 2: Lactic Acid Production by Lb. plantarum in SSF Process with SoAMY-TlGAMY

#### 3.3.1. Flask SSF Cultures

**Figure 5:**
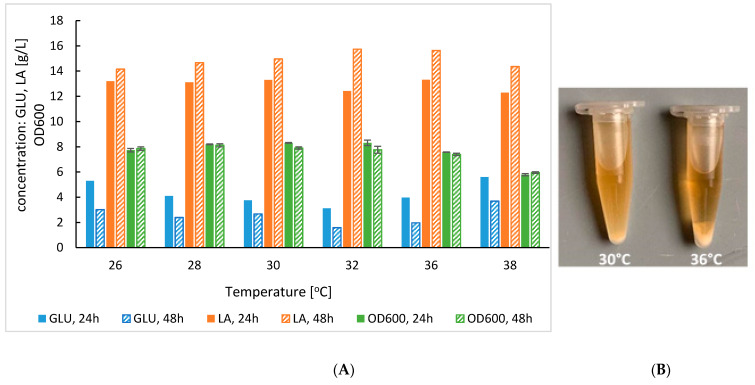
(**A**) Lactic acid production, residual glucose concentration and biomass growth in *Lb. plantarum* cultures conducted on glucose at different temperatures in two time-points (24 h and 48 h). Letter codes: LA, lactic acid; GLU, glucose. (**B**) difference in growth characteristics.

The second exemplary process implementing the SoAMY-TlGAMY preparation was oriented on lactic acid production by *Lb. plantarum* own isolate strain. Prior to the SSF processes, the optimal temperature for *Lb. plantarum* growth and lactic acid production was determined. The results presented in Figure 5. show that the temperature above 32 °C limited the bacteria growth. Growth was the highest and comparable at the temperatures 28, 30 and 32 °C (*p* < 0.05), with its top at 30 °C. Correspondingly, the most rapid lactic acid production was observed at 30°C reaching 13.31 g/L in 24 h (vs. 12.4 at 32 °C and 13.11 at 28 °C, in 24 h). Within this temperature range, residual glucose level was comparable, without significant differences (at *p* < 0.05).

**Figure 6:**
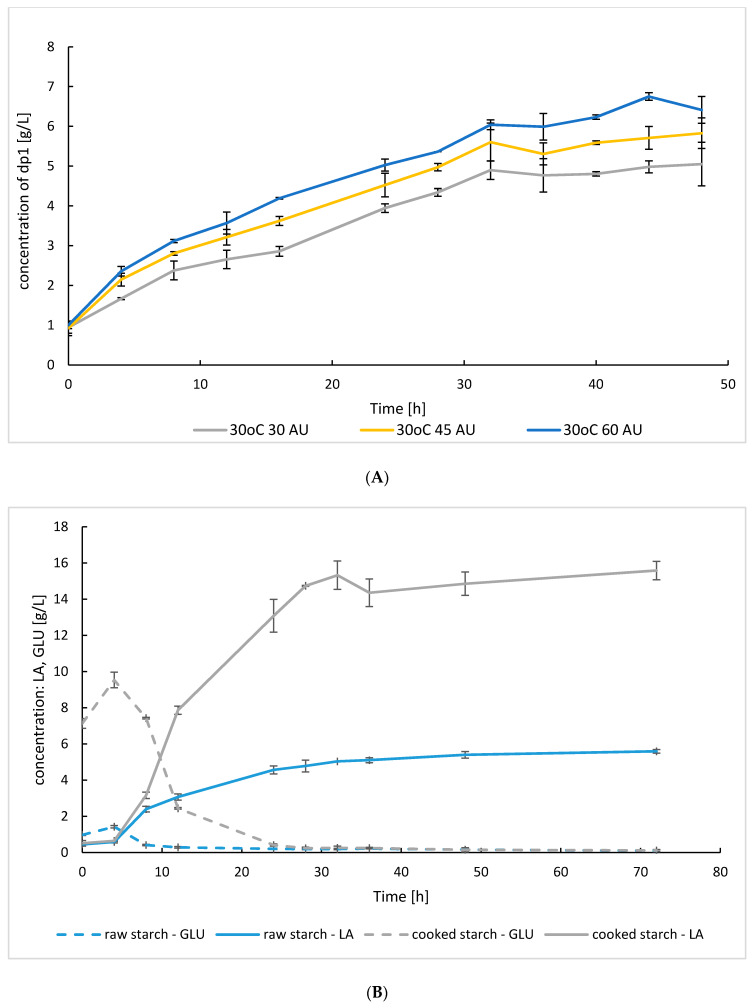
Time course of (**A**) dp1 release at 30 °C by three different doses of SoAMY-TlGAMY preparation; 30, 45 and 60 AU per gram of starch. Time course of (**B**) lactic acid production and utilization of generated glucose by *Lb. plantarum* in SSF process conducted on raw starch and cooked starch supplemented with 60 AU per gram of starch. Letter codes: LA, lactic acid; GLU, glucose. Values indicate means ± SD from three independent runs.

To determine the amount of assimilable sugar released by the SoAMY-TlGAMY crude preparation from the native rice starch under thermal conditions facilitating growth and lactic acid production by *Lb. plantarum*, three doses of the preparation were tested (30, 45, 60 AU per gram of starch) at 30 °C. As presented in (Figure 6A), at this temperature, an increase in preparation dose from 30/45 to 60 AU per gram of starch triggered statistically significant growth in release of assimilable dp1 (*p* < 0.001), reaching 5.15 ± 0.089 g/L at 24 h of hydrolysis for 60 AU per gram of starch.

Based on the initial presumptions, the second proof-of-concept process exploited bacterial strains for the production of lactic acid. The principal difficulty found with these processes was exploitation of raw substrate and inability to apply antibiotic protection against undesired microbiota (as it was the case in processes with *K. marxianus*). Therefore, apart from the typical SSF conducted without thermal pre-treatment of starch, a completely sterilized medium was included in these experiments (“cooked starch” variants). Comparison of the SSF processes conducted with raw starch and cooked starch with *Lb. plantarum* and SoAMY-TlGAMY preparation (60 AU per gram of starch) is presented in Figure 6B. Expectedly, the process with cooked starch was characterized by significantly higher efficiency in terms of lactic acid production (15.58 ± 0.51 vs. 5.60 ± 0.10 g/L at 72 h; *p* < 0.01), which resulted from better accessibility of the cooked biopolymer for SoAMY-TlGAMY enzymes and higher provision of the assimilable carbon (peak 9.54 ± 0.65 vs. 1.43 ± 0.58 g/L at 4 h). Lactic acid production from cooked starch was the most rapid during approximately the first 32 h of culturing, reaching a value of 15.32 ± 0.79 g/L and high lactic acid yield 0.654 g/g at 24 h (Table 2). Afterwards, the process entered stationary phase, probably due to exhaustion of carbon source (starting starch concentration 20 g/L).

#### 3.3.2. Bioreactor SSF Cultures

**Figure 7:**
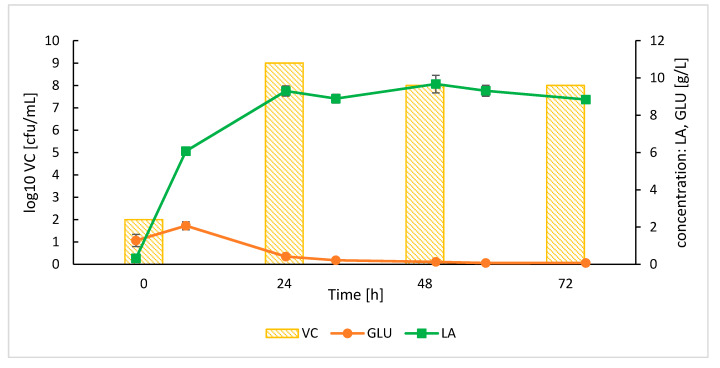
Time course of lactic acid production, residual glucose concentration and viable counts in SSF process on cooked starch with *Lb. plantarum*. Letter codes: LA, lactic acid; GLU, glucose; VC—viable counts. Values indicate means ± SD from two independent runs.

While the trials of lactic acid production by *Lb. plantarum* from raw starch in bioreactor SSF processes were conducted, recurring microbial contaminations directed further efforts towards processes with cooked starch as the main carbon source. Results on lactic acid production and microbial growth in SSF processes with cooked starch, SoAMY-TlGAMY preparation (dose 60 AU per gram of starch) and *Lb. plantarum* are shown in Figure 7.

The lactic acid production rapidly rose during the first 24 h of culturing up to 9.30 ± 0.27 g/L and yield of 0.465 g/g (Table 2), reaching plateau afterwards. The production of lactic acid was corresponding to the cell growth which was also the highest during the first 24 h (10^9^ cfu/mL) and ceased in the following time-points. Glucose consumption rate was higher than its release from starch, as its level was maintained close to zero throughout the process, while starch concentration decreased.

## 4. Discussion

In our previous studies, after cloning and expression of an insect-derived gene encoding alpha-amylase in *Y. lipolytica* [31,32], we evidenced its RSD-capacity, highly desired in starch-based bioprocesses. The production process was scaled-up [38] and the obtained enzyme, supplemented with commercial glucoamylase activity, was applied in an SSF processes with raw starch [39]. To further improve the enzyme’s production by *Y. lipolytica*, synthetic fusions with different signal peptides (SP) were constructed [30]; the best fusions were determined and used in the following studies. Recently, we constructed a panel of *Y. lipolytica* strains expressing the two heterologous genes encoding complementary amylolytic activities alpha-amylase SoAMY and glucoamylase TlGAMY from filamentous fungi [28]. In that previous research, we studied the impact of the expression cassette design and the type of SP on the resultant strain’s amylolytic phenotype. Importantly, the recombinant strains expressing the two genes acquired amylolytic activity, sufficient to support growth in media containing starch of different plant origin (rice, corn and potato) in raw and thermally pre-treated form. Medium used for those studies was largely limited in nutrients from complex components (yeast extract and peptone; 10-fold vs. typical composition) to reliably study growth on starch as the main carbon source. While this strategy was useful for reliable examination of the starch hydrolyzing activity and potential usefulness of the obtained strains as consolidated biocatalysts, it definitely limited the production of the heterologous enzymes.

In the present study, we used the most efficient amylolytic strain from that previous set of the SoAMY-TlGAMY-producing strains, with the aim to use it as a microbial cell factory of RSD amylolytic preparation composed of two enzymes. The strain GGY215 (SP3-G1TG2S) was cultivated in fed-batch bioreactor cultivations in rich medium, to promote production of the enzymes. The cultivation protocol largely relied on our previous experience with single protein-producing strains [25,38] in terms of medium composition and cultivation parameters, however, this time we followed one-step feeding strategy by [24], which is much simpler, but at the same time operable and efficient. The proteins were then precipitated and partly purified. The adopted procedure enabled 10-fold purification factor (based on specific activities of the extracellular SoAMY-TlGAMY proteins in the crude preparation and the post-culturing medium), which is satisfactory and comparable to previous reports. The same purification factor was obtained after size-exclusion chromatography conducted without prior precipitation [40]. Slightly lower value, of 7-fold purification, was achieved after ethanol precipitation of RSDE [16]. Purification to apparent homogeneity, through the applied here affinity chromatography, was facilitated by affinity tags transcriptionally fused to both amylases at C-termini. While it indeed greatly improved the purification factor of the preparation (to over 1500-fold based on specific activities in F2 and AS pellet fractions), it was accompanied by high losses of the enzymes, and thus was not continued.

The concentrated and partly purified preparation SoAMY-TlGAMY was then tested in the SSF processes carried out in the media with starch as the main carbon source with the thermotolerant *K. marxianus* DSMZ 5422 strain for the production of ethanol, and with the *Lb. plantarum* own isolate for the production of lactic acid. These processes were conducted as a proof-of-concept that application of the SoAMY-TlGAMY crude preparation supports sufficient starch hydrolysis enabling the microbial growth and production of the targeted molecules as the selected strains were confirmed to lack amylolytic activity. The crude preparation was also tested for the profile of oligosaccharides released from starch after treatment. Notably, saccharides from dp1 to dp7 were tested, however, only dp1 (glucose) (out of the analyzed range) was present at detectable level in the post-reaction mixtures. Such an outcome was expected, as previously determined dp profile for SoAMY amylase solely was also dominated by glucose [39]. It cannot, however, be excluded that oligosaccharides of higher polymerization degree were present in the post-hydrolysis mixture, but were not analyzed in this and the previous study. 

Compromising thermal optima for enzymatic and microbial catalysts action is the key, initial challenge in developing a new SSF process. As previously shown, the SoAMY alpha-amylase contained in the preparation exhibits maximum activity at 40 °C [32]. The glucoamylase component, TlGAMY was previously reported to have its thermal optimum at 60°C or higher [41]. However, our own tests with *Y. lipolytica*-produced TlGAMY [30] indicated lack of significant difference in its activity between 40 and 60 °C (not shown). For *K. marxianus*, the cultivation temperatures (32, 36 and 40 °C) were chosen based on literature data, suggesting a range between 30 and 40 °C [42], or 40 °C as optimum for ethanol production in SSF process [43,44,45]. Here, obtained data indicated that the ethanol production in the SSF process was the highest at 36 °C (Figure 3B), which was in the middle of range previously adopted SSF temperatures starting from 34 °C [46] to 42 °C [47,48]. This temperature was adopted in the following bioreactor cultivations. For the second strain used in this study, *Lb. plantarum*, the reported optimal temperatures are highly variable, starting from 30 °C [49], through 35 °C [35] up to 45 °C [50]. Here, obtained results indicated that increasing the temperature above 32 °C triggers undesired decrease in the growth and lactic acid synthesis by the used *Lb. plantarum* strain. As shown in Figure 5, the type of microbial growth in liquid medium was changed between 30 °C and >32 °C from turbid, cloudy to sediment. Since at 30 °C, the lactic acid production was the most rapid (peak in 24 h), this temperature was chosen for further studies; in correspondence to previous results obtained by [49].

Simultaneously with the investigation into the optimal growth temperature for the two strains to be used in the proof-of-concept processes, different doses of the crude SoAMY-TlGAMY preparation were tested. Starting doses were established based on previous reports on the SoAMY and TlGAMY [32,41] compared with the total amylolytic activity of here obtained crude preparation. Since the *K. marxianus* can grow at higher temperatures, close to the optimum for the enzymes (40 °C), lower doses were tested in this temperature range (20 and 25 AU per gram of starch). Due to growth limitation of *Lb. plantarum* at >32 °C, the cultivations had to be conducted at 30 °C, and thus higher doses of the preparation were tested in these temperatures (30, 45 and 60 AU per gram of starch). Expectedly, for the *K. marxianus* processes, higher doses of the enzymatic preparation gave higher concentration of produced ethanol by generating higher levels of fermentable sugars (Figure 3A), but those differences were not statistically significant. As such, a lower dose of 20 AU per gram of starch was considered as reasonable for efficient ethanol production. Correspondingly, for the process with *Lb. plantarum* conducted at 30 °C, an increase in the preparation dose to 60 AU brought significant improvement in released dp1 (*p* < 0.05). Since in this process further manipulation with cultivation temperature, enabling lowering the preparation doses, was not possible, a higher dose of the preparation was chosen for *Lb. plantarum* cultivations on starch.

During the *Lb. plantarum* SSF process development, recurring contaminations with undesired microbiota from raw-starch, and inability to provide antibiotics protection, made it reasonable to conduct trials with sterilized substrate. Improved accessibility of autoclaved starch to the amylase’s activity significantly improved efficiency of the process (15.58 ± 0.50 vs. 5.59 ± 0.10 g/L lactic acid) and prevented contaminations (routinely checked by microscopic observations). Notably, studies on SSF with *Lactobacilli* spp. are conducted on heat-pre-treated (autoclaved) soluble starch rather than raw substrate e.g. [35,51,52,53]. Here obtained lactic acid concentration by the *Lb. plantarum* in the presence of the SoAMY-TlGAMY was close to the results reported for *Lb. plantarum* grown on cassava starch (9.67 and 10.34 g/L, respectively) [50]. The other reports on *Lactobacilli* spp. exploitation in the SSF processes were conducted at higher substrate load, up to 150 g/L of starch [54], which gave higher LA yield (0.867 g/g).

SSF processes with the *K. marxianus* and the SoAMY-TlGAMY preparation (36 °C, 20 AU per gram of starch) could be conducted with raw, non-thermally pre-treated starch, and thus—fully exploit potential of the SoAMY-TlGAMY RSD preparation. Upon developing the proof-of-concept process we much focused on establishing appropriate aeration strategy. It is known that under aerobic conditions, ethanol synthesis is blocked, but also *K. marxianus* synthesizes large amounts of volatile compounds, namely ethyl acetate, resulting in losses of ethanol yield [55,56]. In the present study, the ethyl acetate concentration was tracked during cultivations, however, each time it was detected at trace amounts (results not shown), suggesting that oxygen provision was not too high. Silveira et al. [57] analyzed the ethanol production by *K. marxianus* under three aeration condition (aerobic, hypoxic and anoxic) and showed that maximum ethanol yield was obtained under anoxia, followed by hypoxia. In contrast, Kuloyo et al. [43] showed that in the oxygen-limited cultures, the ethanol productivity was almost double that obtained in the non-aerated cultures, evidencing that small level of oxygen is necessary to promote the yeast cell growth and by this, increase titer of ethanol. Similar conclusions could be withdrawn from our data, where either IS21 or HS aeration strategy enabled higher production of ethanol by the *K. marxianus* strain. The maximum ethanol concentration in the *K. marxianus* SSF cultures on raw starch reached 12.11 ± 0.26 g/L (Figure 4A). When compared with the other literature reports using comparable substrate load (< 10%), here obtained titers are comparable to 14 g/L achieved under oxygen-limited conditions in [43], but is also 2-fold lower than the ethanol concentrations reported in [58,59]. In the other studies, when a higher load of the substrate was implemented, the ethanol concentration reached 49 g/L [44], 80 g/L [57] or even 110 g/L, obtained in our previous studies [8]. The highly increased substrate load and different processing conditions were used in those studies, which makes direct comparison not fully reliable. However, the solutions proposed in those studies should be considered in further optimization of processes implementing the SoAMY-TlGAMY preparation.

Finally, efficiency of the *K. marxianus* SSF process in raw starch with the SoAMY-TlGAMY preparation was evaluated in comparison to three reference cultures (Figure 4B). When compared with the control culture conducted on glucose as the main carbon source, the maximum ethanol titer reached 77% of the control (10.02 ± 0.19 to 13.59 ± 0.27 g/L in SSF SoAMY-TlGAMY and control G, respectively). This observation suggests that indeed application of the SoAMY-TlGAMY preparation gives satisfactory starch hydrolyzing activity for the ethanol production from raw starch. In contrast, the reference culture conducted with commercial RSD preparation STRAGEN gave 2-fold higher ethanol titer than the one obtained with SoAMY-TlGAMY (21.22 ± 1.95 vs. 10.02 ± 0.19 g/L). The previous studies on comparison of the commercial STARGEN preparation and the SoAMY-containing amylolytic preparation supplemented with commercial glucoamylase (to reach the same amylolytic AU per gram of starch) indicated lack of significant differences in the cultures performance in terms of ethanol production [39]. Comparison of these two outcomes suggests low efficiency of TlGAMY as the glucoamylase component in the SoAMY-TlGAMY preparation; since when TlGAMY was substituted with a commercial glucoamylase, the preparation performed equally well as the commercial RSDE. However, it has to be stated that specific, individual contribution of the alpha-amylase and the glucoamylase activity in the novel preparation was not evaluated, but only total amylolytic activity. Hence, such conclusion is unsupported by the current data, and further insight in this issue is needed. Notably, the reference culture with the commercial preparation conducted on raw starch, resulted in higher ethanol titer than that obtained in the control culture on glucose, despite similar glucose utilization and cell growth profiles. It is presumed that the STARGEN preparation continued to release the fermentable sugars from the raw starch throughout the culturing time, so ultimately provided more carbon source for the cell growth and the ethanol production. This was missed in the HPLC analysis, as the cells continued to utilize any glucose immediately upon its release. Due to favorable conditions, the assimilable carbon was completely transformed into ethanol, as the biomass growth curve were corresponding for these two culture variants. Finally, as expected comparison of SSF and SHF processes showed that higher ethanol titer could be reached in the SSF process, as evidenced in the other studies [43,44,60,61], demonstrating superiority of the SSF process over the SHF process.

## 5. Conclusions

In summary, this study falls into a general trend of exploiting microorganisms as cellular factories providing the market with desired biomolecules. Here we used *Y. lipolytica* as a heterologous protein expression platform to produce the RSD-amylolytic preparation composed of two enzymatic activities: alpha-amylase SoAMY and glucoamylase TlGAMY. The partly purified preparation was then used in the two proof-of-concept processes operated according to the SSF strategy, exploiting microbial biocatalysts devoid of amylolytic activities in their native form. The thermotolerant *K. marxianus* was used for the production of ethanol, while the *Lb. plantarum* strain was used for the lactic acid formation. Depending on the used microorganisms, the two SSF processes were subjected to different limitations and bottlenecks, and thus the optimization studies were focused on different aspects. The principal limitation encountered with *Lb. plantarum* was inability to operate with raw, non-sterilized substrate, which caused recurring contaminations. Additionally, the thermal growth optima for the LAB strain was low in relation to the thermal optimum of the RSD-preparation activity. Altogether, these factors forced application of large quantities of the preparation and operation in thermally pre-treated starch, which was not desired. On the other hand, the thermotolerance of *K. marxianus* and possibility of implementing antibiotic protection, to eliminate undesired microbiota, allowed to fully exploit the RSD potential of the new preparation in the ethanol production process. After insight into oxygen control strategy, the process with the novel RSD-preparation was compared with the process conducted with commercial preparation, full availability of assimilable sugars and the SHF process. Comparisons with the SHF process and the control run conducted on glucose showed that the SSF with SoAMY-TlGAMY preparation on raw starch holds promise of efficient process, after further optimization. Nevertheless, as evidenced by a process conducted with the commercial preparation, further insightful studies are needed.

## Figures and Tables

**Table 1:** Amylolytic activity, total protein concentration and specific amylolytic activity of protein fractions: supernatant after the ammonium sulfate precipitation and centrifugation (AS supernatant), resuspended protein pellet after the ammonium sulfate precipitation and centrifugation (AS pellet), fractions obtained during the affinity chromatography: flow-through (F-T), wash-unbound (W-U), first and second fraction with increased Abs280 nm absorbance (F1, F2). Presented values give means from technical triplicate ±SD for a single control fed-batch run. * ±SD from technical triplicate conducted for four independent fed-batch culture runs.

Protein Fraction	Activity [AU/mL]		Total Protein [mg/mL]		Specific ACT [AU/mg]	
	AU/mL	±SD	mg/mL	±SD	AU/mg	±SD
AS supernatant	0.18	0.16 *	4.12	1.31 *	0.06	0.05 *
AS pellet	317.83	21.57 *	40.22	4.83 *	8.01	1.40 *
F-T	64.05	0.92	26.67	3.41	2.40	0.03
W-U	328.94	14.62	4.08	0.17	80.65	3.58
F1	366.19	10.50	0.74	0.47	493.30	14.14
F2	5609.29	199.70	0.43	0.01	13159.51	468.49
Purification factor F2 vs. AS pellet [fold]						1642.97×

**Table 2:** Yield and concentration of ethanol and lactic acid in flask and bioreactor SSF (simultaneous saccharification and fermentation) cultivations of *K. marxianus* and *Lb. plantarum* on raw (or cooked for *Lb. plantarum*) starch with SoAMY-TlGAMY preparation. Due to difficulties in assessing residual starch concentration in raw starch cultures, the yield values were expressed as a gram of product produced per gram of the substrate provided at the beginning of culturing, according to [37]. All the values were calculated for 24 h time-point of the cultures. Abbreviations: IS: air provision by immersed sparger, HS: air provision to headspace, IS21: air provision by immersed sparger, for the first 21 h, control G: control with glucose as a substrate, STARGEN: control with commercial preparation, SHF: separate hydrolysis and fermentation.

Culturing Variant	Yield [g/g of Provided Substrate] at 24 h	Product Concentration (g/L) at 24 h
EtOH Production by *K. marxianus* in SSF		
*Flask*		
32 °C 20 AU	0.179	7.18
32 °C 25 AU	0.196	7.86
36 °C 20 AU	0.177	7.09
36 °C 25 AU	0.193	7.73
40 °C 20 AU	0.158	6.34
40 °C 25 AU	0.191	7.66
*Bioreactor*		
IS	0.144	5.76
HS	0.192	7.68
IS21	0.173	6.91
control G	0.340	13.59
STARGEN	0.515	20.59
SHF	0.164	6.561
**Lactic acid production by *Lb. plantarum* in SSF**		
*Flask*		
30° 60 AU raw starch	0.228	4.570
30° 60 AU cooked starch	0.654	13.079
*Bioreactor*		
30° 60 AU cooked starch	0.465	9.301
